# Use of machine learning for early prediction of short-term mortality in veterans with metabolic dysfunction-associated steatotic liver disease

**DOI:** 10.1371/journal.pone.0334715

**Published:** 2025-10-27

**Authors:** Lewis J. Frey, Michael Fuchs, Ralph C. Ward, Mulugeta Gebregziabher, Ahmad Basil Nasir, Yamini Natarajan, Andrew Schreiner, Don C. Rockey, Wing-Kin Syn

**Affiliations:** 1 Ralph H. Johnson VA Medical Center, Charleston, South Carolina, United States of America; 2 Saint Louis University, St Louis, Missouri, United States of America; 3 Hunter Holmes McGuire VA Medical Center, Richmond, Virginia, United States of America; 4 Virginia Commonwealth University, Richmond, Virginia, United States of America; 5 Medical University of South Carolina, Charleston, South Carolina, United States of America; 6 Kelsey-Seybold Clinic, Houston, Texas, United States of America; 7 Department of Physiology, Faculty of Medicine and Nursing, University of Basque Country UPV/EHU, Leioa, Vizcaya, Spain; Universita degli Studi della Campania Luigi Vanvitelli Scuola di Medicina e Chirurgia, ITALY

## Abstract

**Background:**

Metabolic dysfunction associated steatotic liver disease (MASLD) is a leading cause of chronic liver disease worldwide and affects >25% in the United States population. We hypothesized that clinical features present in electronic health records (EHR) could be extracted early to characterize patients with MASLD who are at high risk of early mortality and that machine learning models would predict mortality better than noninvasive assessments of liver disease/fibrosis.

**Methods:**

Using previously published criteria for MASLD, applied to data from the US Veterans Affairs EHR, we identified a cohort of 13,071 patients between 2000 and 2018 who had an initial diagnosis of MASLD without clinical evidence of cirrhosis. We subsequently used machine-learning and conducted analysis of variance and logistic regression to identify clinical variables to characterize cirrhosis risk and predict mortality within the ensuing 5-years.

**Results:**

The average age of the cohort was 60 years, had a BMI of 31, and 34% diabetes prevalence. Patients who progressed to cirrhosis were younger when first diagnosed with MASLD (56), had a higher BMI (33), and had significantly higher noninvasive fibrosis scores. Having diabetes at index MASLD diagnosis significantly increased the risk of developing cirrhosis and doubled the risk cirrhosis plus HCC (2.09 CI:1.217–3.63). Our machine-learning model performed significantly better than FIB-4 at predicting mortality within 5-years of being diagnosed with MASLD (AUC 83% vs 68%).

**Conclusion:**

Our data suggest that machine learning models based on data extracted from the EHR early during MASLD can identify patients likely to develop cirrhosis and predict short term mortality.

## Introduction

Metabolic dysfunction associated steatotic liver disease (MASLD)) is a significant and growing health problem that affects greater than 25% of the general population and is strongly associated with the metabolic syndrome (obesity, type 2 diabetes mellitus (T2DM), hypertension, and dyslipidemia) [1]. Up to 20% of those with MASLD may progress to Metabolic dysfunction associated steatohepatitis (MASH) [2], the more advanced stage of disease, which then puts them at risk of developing liver fibrosis, cirrhosis, and cirrhosis-associated complications such as hepatocellular carcinoma (HCC) and liver failure.

Liver enzymes, alanine aminotransferase (ALT) and aspartate aminotransferase (AST), are routinely used by primary care providers (PCP) to identify those likely to have MASH or MASH fibrosis/ cirrhosis (or HCC), but those measures have low sensitivity (i.e., true positive rate) and specificity (i.e., true negative rate), and those with normal ALT levels can span the range of disease severity in MASLD/ MASH (with or without fibrosis/cirrhosis) [3,4]. As a result, patients at high risk for progression to advanced disease stages (MASH, fibrosis, cirrhosis, and HCC) are often overlooked or misdiagnosed, while those with low risk for progression may conversely, be referred for treatment [5]. In recent years, simple noninvasive scores such as the fibrosis-4 (FIB-4) score [6,7], aspartate aminotransferase to platelet ratio index (APRI) [8], and NAFLD Fibrosis Score (NFS) [9], as well as more complex and commercially available tests such as the Fibrotest^®^, Fibrosure^®^, and enhanced liver fibrosis (ELF^®^) panel, have been developed with the aims of improving the detection of fibrosis. While all these tests generally exhibit excellent negative predictive values at cutoff extremes, they remain limited overall by their low positive predictive values, sensitivities, and accuracies.

The aim of this study was to examine readily available clinical features present in electronic health records to identify patients with MASLD who are at risk of early mortality. Moreover, we aimed to develop machine learning models to predict mortality that perform better than established noninvasive measures.

## Methods

### Data sources

We utilized data from the VA Informatics and Computing infrastructure (VINCI) to obtain a cohort of US Veterans. This includes both VA and VA/CMS data sources. We analyzed data from VA National Data Systems (NDS) and from the VA Information Resource Center (VIReC) using VINCI between 2/27/2019 and 2/26/2024. The authors did not have access to information that could identify individual patients during or after data collection. The Medical University of South Carolina (MUSC) institutional review board and the Ralph H. Johnson VA Research and Development Committee approved this study.

### Dataset

We examined a cohort of Veterans meeting previously published criteria for MASLD between January 1, 2000 and January 1, 2018 and having a liver biopsy with at least 5 years of follow up or death within 5 years after first meeting Husain criteria [10]. The group was assessed for eligibility through no concurrent liver disease (ICD-9/10 code for hepatitis B virus (HBV), hepatitis C virus (HCV), alcoholic liver disease, autoimmune hepatitis, biliary cirrhosis, primary sclerosing cholangitis, disorders of iron metabolism, Wilson’s disease), or history of significant alcohol consumption (by ICD-9/10). Using previously published methodology [10], we identified Veterans in the cohort with MASLD using the ICD-9/10 codes, 571.8 and K76.0. Those without an ICD-9 code for MASLD were classified as having MASLD if they had at least two or more elevated ALT values (≥40 IU/ml) more than 6 months apart, with no evidence of positive serologic testing for HBV (HBV surface antigen) or HCV (HCV RNA), and no alcohol related ICD-9/10 codes or positive AUDIT-C scores within one year of elevated ALT. The VINCI platform contains information from annual AUDIT-C screen for alcohol use disorders, which has been used to screen over 90% of VA outpatients nationwide [11]. A score of ≥ 4 was used to identify active alcohol use in men and ≥3 was used in women [2]. Patients with cirrhosis or HCC at index MASLD diagnosis were excluded.

### Variable definitions

#### Outcomes variables.

Cirrhosis: Cirrhosis was identified by the presence of a combination of ICD-9/10 codes for cirrhosis as previously described [12]. Veterans within the study cohort were considered to have cirrhosis if they were determined to meet these published criteria for cirrhosis using data from their medical records.

Hepatocellular carcinoma: The presence of hepatocellular carcinoma was assessed by ICD9/10 codes 155 and C22.0, respectively.

Disease Outcome Grouping: Patients were assigned to one of four groups according to the presence of cirrhosis and/or HCC as follows: those with no evidence either of cirrhosis or HCC (*No CIRR/No HCC (control group))*; those with cirrhosis but no evidence of HCC (*CIRR*); cirrhosis and HCC (*CIRR-HCC*); HCC without evidence of cirrhosis (*HCC*).

Mortality: Death was captured from the VA Vital Status file, which applies an algorithm to a combination of the BIRLS Death File, the Social Security Administration Death Master File, the Medicare Vital Status File and the VHA Medical SAS Datasets.

#### Predictor variables.

Clinical data within 1 year of the diagnosis of MASLD were used for this analysis. We use the term MASLD throughout but cite older literature using Non-Alcoholic Fatty Liver Disease (NAFLD). Fibrosis was assessed using the following noninvasive fibrosis measures: FIB-4 [6,7], NAFLD Fibrosis Score (NFS) [9], aspartate aminotransferase and alanine aminotransferase ratio (AAR), age-platelet index (AP) [13], APRI [8], and a composite of body mass index (**B**MI), A**AR** and **d**iabetes (BARD) score, which is the sum of binary values: 1 point for BMI ≥ 28, 2 points for A**A**R ≥ 0.8, and 1 point for diabetes [14]. Complete demographic information including age, gender, and race/ethnicity as well as clinical data were captured (comorbidities occurring prior to the diagnosis of MASLD such as diabetes, hypertension and dyslipidemia were collected using ICD9/10 codes).

#### Analysis methods.

Descriptive analysis: For the four groups, mean, standard error (SE) and n are reported for the following quantitative variables: age, BMI, FIB-4, NFS, AAR, AP and APRI (Table 1). SAS was used to conduct analysis of variance (ANOVA) for each quantitative dependent variable with group as the independent variable. The significance level was set to p < 0.05. All six pairwise comparisons were examined with Bonferroni correction for multiple comparisons. Multinomial logistic regression was used for dichotomous variables of gender, race, diabetes, hypertension, dyslipidemia and cumulative logistic regression was used for BARD. A multinomial regression analysis was performed comparing the CIRR, CIRR with HCC and HCC groups against the control group (No CIRR/No HCC). We assessed for collinearity and examined correlation of variables > 0.8 and variance inflation (VIF) through checking if VIF > 10. We removed variables from the multinomial analysis if they had collinearity that would impact model fitting.

**Table 1:** Demographics, clinical features, and noninvasive fibrosis assessment.

	Overall (n = 13,071)	No CIRR/No HCC (n = 11,699)	CIRR (n = 955)	CIRR-HCC (n = 140)	HCC (n = 277)
**Age (years)**	59.9 ± 0.1	60.0 ± 0.1^a,b,c^	56.4 ± 0.4^d,e^	63.4 ± 0.8^f^	66.0 ± 0.6
**Male (%)**	93.3	93.3^a,b,c^	91.2^d,e^	99.3	97.1
**BMI (kg/m2)**	31.0 ± 0.1	30.8 ± 0.1^a,b^	33.4 ± 0.2^e^	33.2 ± 0.5^f^	31.0 ± 0.4
**Race/Ethnicity (%)**					
**NHW**	77.4	76.9^a,c^	80.7	83.6	84.1
**NHB**	13.2	13.8^a,b^	8.1	4.3^f^	10.8
**Hispanic**	6.8	6.7	8.0	8.6	4.7
**Other**	2.6	2.6^c^	3.2^e^	3.6^f^	0.4
**FIB-4**	1.6 ± 0.04	1.5 ± 0.03^a,b,c^	2.3 ± 0.37	2.5 ± 0.13	2.1 ± 0.10
**NFS**	−0.78 ± 0.01	−0.87 ± 0.01^a,b,c^	−0.08 ± 0.05^d,e^	0.45 ± 0.13^f^	−0.35 ± 0.09
**AAR**	0.72 ± 0.003	0.71 ± 0.003^a,b,c^	0.75 ± 0.01^d,e^	0.88 ± 0.03	0.84 ± 0.03
**AP**	4.6 ± 0.02	4.5 ± 0.02^a,b,c^	4.9 ± 0.07^d,e^	5.9 ± 0.18^f^	5.4 ± 0.11
**APRI**	0.50 ± 0.01	0.47 ± 0.01^a,b^	0.80 ± 0.11^e^	0.76 ± 0.04	0.57 ± 0.07
**BARD 0**	16.1	17.0^a,b,c^	8.8^d^	4.3^f^	9.4
**1**	37.8	38.5	33.0	20.7	29.6
**2**	25.4	25.0	28.3	29.3	30.3
**3**	12.5	12.4	14.1	15.0	13.0
**4**	8.2	7.1	15.8	30.7	17.7
**Diabetes**	34.3	32.8^a,b,c^	44.1^d,e^	62.9^f^	52.0
**Hypertension**	71.1	70.8^b,c^	70.1^d,e^	78.6	83.0
**Dyslipidemia**	68.8	69.6^a,b^	61.6	57.9	65.0

The superscripts indicate significant pairwise differences.

^a^No CIRR/No HCC and CIRR ^d^CIRR and CIRR-HCC

^b^No CIRR/No HCC and CIRR-HCC ^e^CIRR and HCC

^c^No CIRR/No HCC and HCC ^f^CIRR-HCC and HCC

Feature Selection: A feature ranking algorithm was run to order features in a list from most relevant to least at predicting mortality on the training data. We used a gradient tree boosting machine model for our feature ranking analysis through the application of extreme gradient boosting (XGBoost) to run the regression trees to predict mortality [15]. We used a unified framework for interpreting the impact of features on the predictions based on Shapley additive explanations (SHAP) that generated a ranking of features using SHAP values [16].

Starting with the highest ranked features, the features were incrementally added to a random forest classifier to determine if they improved classification performance. Random forests use a collection of tree-structured classifiers where each tree casts a vote for the most popular class given the input vector. If a feature improved performance, it was selected to be part of the model and the next highest ranked feature was added to the model and assessed for performance improvements. If a feature did not improve performance, it was excluded from the model.

Predictive Analysis: We compared random forest classifiers with feature selection to FIB-4 as this is widely used to estimate risks of advanced fibrosis/ cirrhosis, HCC, and mortality [7,17]. We used the python xgboost package to run XGBoost for feature ranking by SHAP values, the sklearn package to run the random forest classifier for predicting the classes of instances using default hyperparameters unless other values are specified, and lifelines for the Kaplan Meier analysis. The following binary predictions are compared: whether Veterans died within five years when criteria for MASLD were first present (survived n = 10,665, died n = 2,406). The receiver operator characteristic (ROC) curve was used to calculate the area under the curve (AUC) to compare performance across models. The random forest classifier was trained on a balanced training set composed of two thirds of the minority category and an equal number randomly selected from the majority category. The test set consisted of the remaining holdout data from both categories. We assessed predictions on the holdout test sets for both FIB-4 and random forest for each of the mortality predictions to generate the ROC curves. SAS System Version 9.3 (Cary, North Carolina) was used to conduct statistical analyses. Unless stated otherwise, alpha is set to 0.05.

## Results

**Fig 1:**
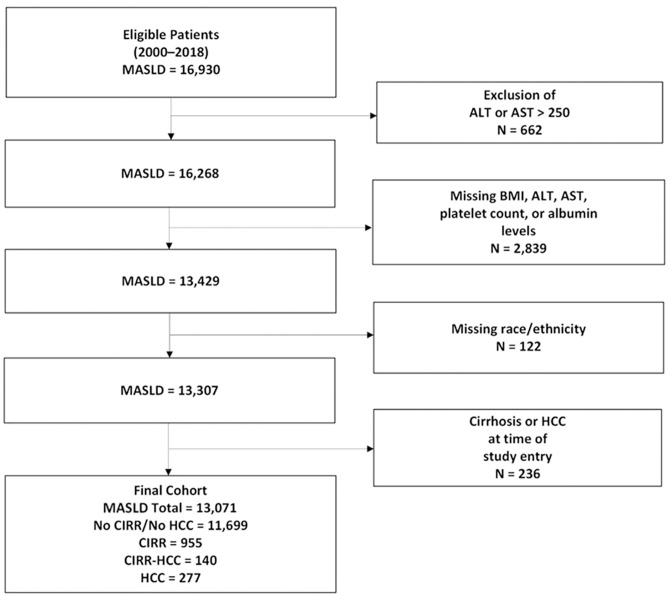
Flow diagram for the study.

A total of 16,930 patients were identified as meeting criteria for MASLD. Patients with the following features were excluded: ALT or AST > 250 (n = 662), missing BMI, AST, ALT, platelet count, or albumin levels (n = 2,839), missing race/ethnicity (n = 122), and codes for cirrhosis or HCC at the time of study entry (n = 236) (**Fig 1**). The exclusion of AST or ALT greater than 250 is to exclude acute hepatitis flares or other liver diseases not representative of chronic MASLD. Patients with incomplete data for BMI, AST, ALT, platelet counts, race, or ethnicity were excluded from the final analysis, and no data imputation was conducted. Clinical data within 1 year of the diagnosis of MASLD were used for this analysis (and on average were within one month). This provided a snapshot of disease characteristics at the time of first MASLD diagnosis.

The overall cohort was primarily older white males (**Table 1**). The mean age associated with first diagnosis of MASLD was significantly older for the CIRR-HCC and HCC groups and significantly younger for the CIRR group compared to control (**Table 1**). All groups were obese (BMI > 30 kg/m^2^) with a higher BMI for those with cirrhosis compared with control. There were significantly fewer non-Hispanic black (NHB) with CIRR and more non-Hispanic white (NHW) with CIRR compared with control. Diabetes was significantly associated with CIRR, CIRR-HCC and HCC compared with control. Hypertension was significantly higher in CIRR-HCC and HCC compared with control. Dyslipidemia was higher in the control group compared with CIRR and CIRR-HCC. At the time of first MASLD diagnosis, FIB-4, NFS, AAR, AP, and BARD were significantly higher in CIRR, CIRR-HCC, and HCC groups compared with the control. APRI was significantly higher for CIRR and CIRR-HCC. Based on noninvasive tests of fibrosis, patients with CIRR-HCC had the most liver fibrosis.

### Multinomial regression analysis

Multinomial regression demonstrated that CIRR patients were younger and HCC patients were older than control group patients when first meeting MASLD criteria (**Table 2**). NHB were half as likely as NHW of being in the CIRR group and one-third as likely of being in CIRR-HCC. Women were one-tenth as likely to be in the CIRR-HCC group. Having diabetes when criteria for MASLD were first present significantly increased the risk of CIRR, CIRR-HCC, and HCC, doubling the risk of CIRR-HCC. Dyslipidemia was more likely in the control group when first meeting MASLD diagnosis criteria compared with the other groups. BARD values were significantly higher than control for the CIRR and CIRR-HCC groups. The variable APRI had a correlation of 0.8 with FIB4 and was excluded from the model. All other variables met the correlation and VIF cutoffs.

**Table 2:** Multinomial Regression Analysis of features associated with CIRR, CIRR-HCC or HCC.

	CIRR	CIRR-HCC	HCC
**Age**	0.937* (0.928 - 0.947)	0.984 (0.961 - 1.008)	1.033* (1.016 - 1.050)
**Sex**	1.085 (0.844 - 1.394)	0.108* (0.015 - 0.784)	0.569 (0.278 - 1.166)
**Black Non-Hispanic**	0.502* (0.391 - 0.644)	0.294* (0.128 - 0.674)	0.721 (0.487 - 1.400)
**Hispanic**	1.084 (0.839 - 1.400)	1.383 (0.751 - 2.546)	0.703 (0.398 - 1.241)
**Other**	1.155 (0.783 - 1.704)	1.501 (0.599 - 3.761)	0.146 (0.020 - 1.047)
**BMI**	1.039* (1.022 - 1.056)	1.044* (1.002 - 1.087)	1.019 (0.992 - 1.047)
**Diabetes Mellitus**	1.337* (1.070 - 1.672)	2.094* (1.207 - 3.633)	1.882* (1.335 - 2.653)
**Hypertension**	1.024 (0.863 - 1.214)	0.905 (0.574 - 1.425)	1.285 (0.910 - 1.816)
**Dyslipidemia**	0.683* (0.587 - 0.795)	0.353* (0.245 - 0.509)	0.514* (0.394 - 0.670)
**FIB-4**	1.010 (1.000 - 1.020)	1.000 (0.953 - 1.050)	1.000 (0.960 - 1.041)
**NFS**	1.017 (0.901 - 1.148)	0.988 (0.727 - 1.342)	0.943 (0.806 - 1.102)
**AAR**	1.129 (0.838 - 1.521)	1.514 (0.923 - 2.483)	1.569* (1.126 - 2.187)
**AP**	1.346* (1.254 - 1.445)	1.384* (1.167 - 1.640)	1.093 (0.978 - 1.221)
**BARD**	1.210* (1.103 - 1.327)	1.454* (1.197 - 1.766)	1.086 (0.942 - 1.056)

An asterisk (*) indicates significance(p < .05). APRI was not included in the model due to collinearity. Each column lists odds ratios with the 95% confidence intervals in parentheses for the variables for each category with the control group of No CIRR/No HCC used as the reference category.

### Clinical features important in prediction of 5-year mortality

**Fig 2:**
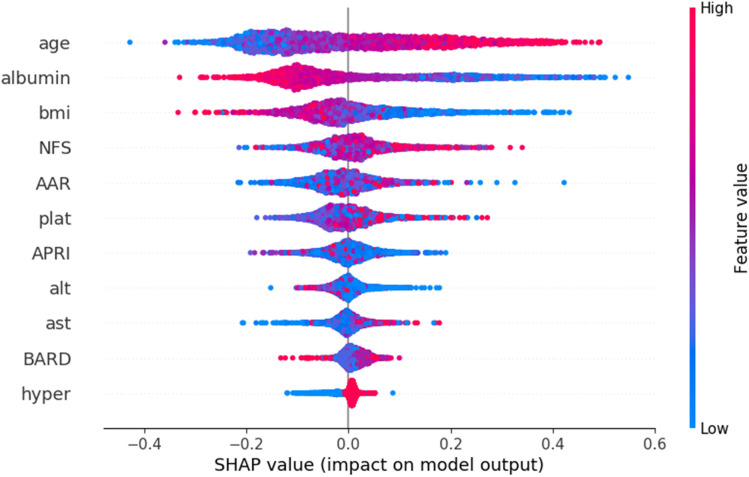
The SHAP values for the selected variables that improve training accuracy in the nested cross validation. SHAP values higher than 0.0 move the classifier to predict death while points with lower SHAP values move the classifier to predict alive. The red color indicates values were high and blue indicates values were low. In the case of age, higher values (red) indicating older patients moved the classifier in the direction of predicting death, while lower values (blue) of albumin moved the classifier to predicting death. The variables considered included age, sex, bmi, race, ethnicity, NFS, AAR, AP, APRI, BARD, alt, ast, diabetes, hypertension, and dyslipidemia.

Selection of relevant demographic, clinical, and noninvasive fibrosis assessment features were ranked by their impact on the training performance of the predictive model (Fig 2). Higher values of age and lower values of albumin and BMI increased the likelihood of the model predicting death within five years. Diabetes increased the probability of predicting death through the composite variable BARD. The ranking of features was determined through an evaluation of performance based on the training set that consisted of 3,224 cases balanced between the majority and minority classes (alive at 5-years n = 1,584 and dead within 5-years n = 1,640). In brief, features are sorted by SHAP relevance values and then incrementally added to a random forest classifier with 1000 trees using only the training data in 5 cross fold split. The features that increase AUC performance are added to the set of features to be used in the final model. The SHAP value plot in Fig 2 has the ranked order of the selected features with age being at the top. The position on the x-axis indicates the degree of impact on the model with red being high values and blue being low values for the feature. The plot shows older age (in red) was associated with increased risk of death and lower albumin values (in blue) also were associated with increased risk of death. The random forest used the selected features from Fig 2 in the final model and is trained on the same training data and evaluated on a holdout test set. The holdout test set consisted of one-third of cases of those who died within 5-years (n = 766) and the remaining cases were those alive at five years (n = 9,081) for a total test set of 9,847 cases.

### Predictive models of mortality

**Fig 3:**
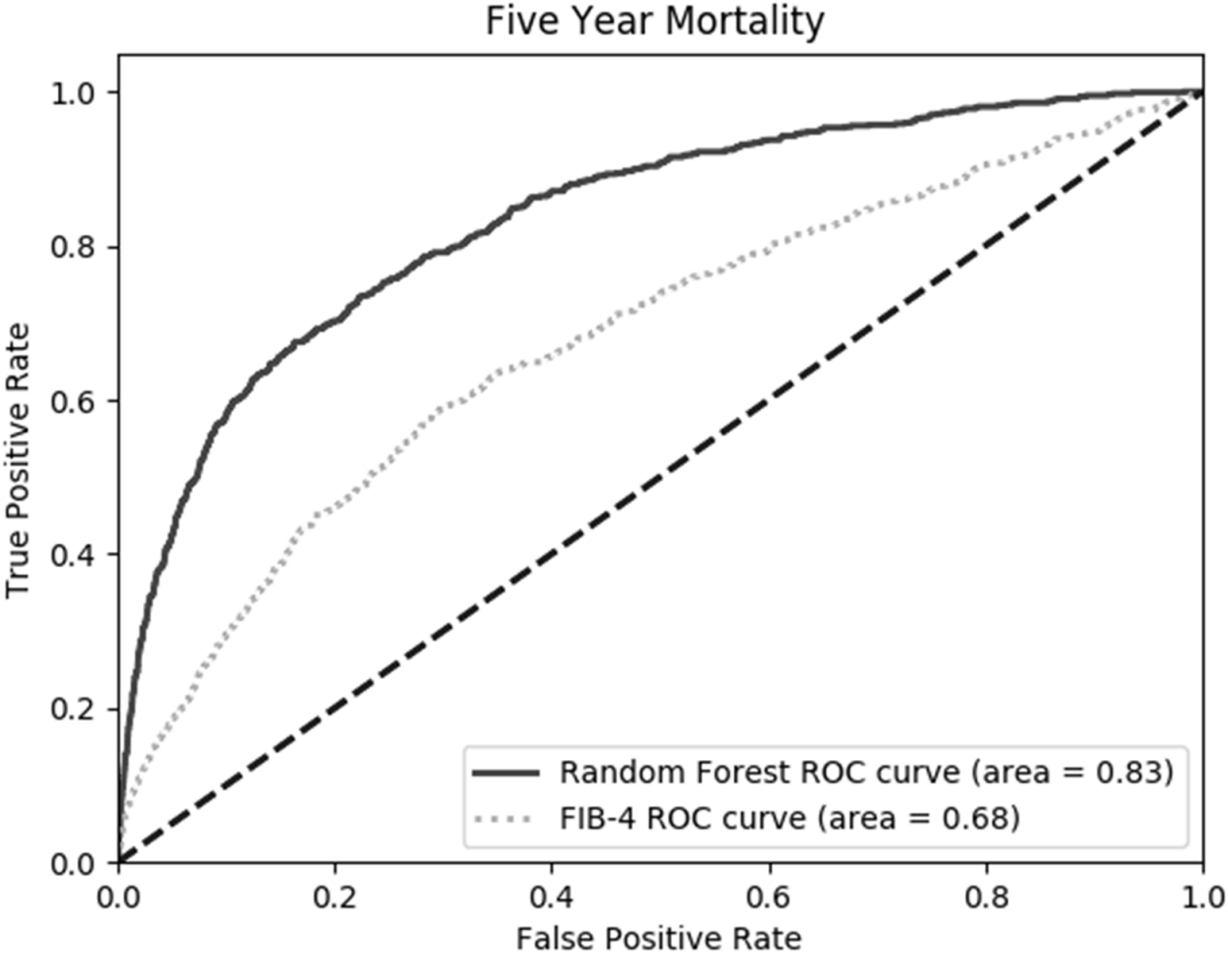
AUC comparison of random forest and FIB-4 at predicting mortality within five years of meeting MASLD criteria on holdout test set. The models are significantly different at p < 0.05 (Chi-Square).

The trained model based on the relevant features from Fig 2 were used for the evaluation of the random forest model on the holdout test set. For predicting mortality within five years of first identification of MASLD, the random forest model (0.83 AUC) was significantly better than FIB-4 (0.68 AUC) due to the higher sensitivity of the random forest (See Fig 3, Table 3). The mortality assessed was all-cause mortality, but the results hold when filtering for only cirrhosis and HCC patients.

**Table 3:** The AUC performance of models along with sensitivity, specificity, positive predictive value (PPV), and negative predictive value (NPV) for the random forest classifier and FIB-4 on the test set. The FIB-4 threshold of 2.67 is used for FIB-4 classification.

	Model	AUC ROC	Sensitivity	Specificity	PPV	NPV
**Mortality**	**RF**	0.83*	**0.71**	**0.79**	**0.22**	**0.97**
	**FIB4**	0.68	0.22	0.93	0.21	0.93

*p < .05 RF vs FIB-4.

**Fig 4:**
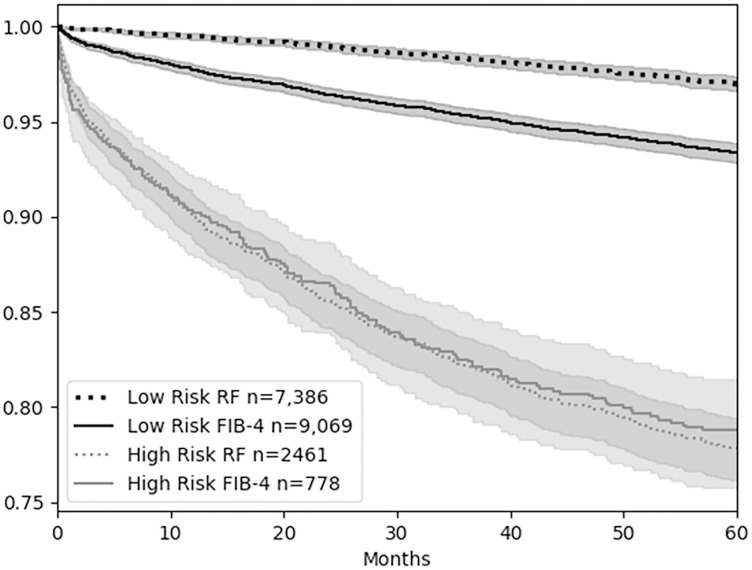
Kaplan Meier plots of random forest (RF) and FIB-4 high and low risk categories on holdout test set. The RF and FIB-4 Low Risk models are significantly different at p < 0.05 (Log-Rank Test), while the High Risk models are not significantly different.

Comparing the Kaplan Meier plots for the low risk groups of random forest and FIB-4 the higher sensitivity of the random forest results in the low risk group having significantly fewer deaths over the five years compared with FIB-4 (See Fig 4). The high risk groups for random forest and FIB-4 overlap and are not significantly different by the Log-Rank Test.

## Discussion

The pathogenesis of MASLD is complex and heretofore, no single clinical measure is sensitive to predict which patients will die within five years of first meeting criteria for having a MASLD diagnosis. Here we have demonstrated that machine learning using random forest with SHAP feature extraction is sensitive and performed better than FIB-4 at predicting mortality after meeting MASLD criteria. The random forest is consistent with predicting those who die early in the five years and those who die later as shown in the Kaplan Meier plot. The FIB-4 low risk model is less consistent over the five years and miss identifies those who will die early in the five year window as low risk. The current study shows that the use of clinical and laboratory measurements in combination help identify individuals with MASLD who are at risk of mortality. The results further indicate that there is information in the EHR that might be utilized to inform early clinical decision making – specifically, the signal in EHR recorded non-invasive measures might be used to prioritize patients for more aggressive screening and/or evaluation. The approach moves the predictions to early MASLD diagnosis and extends the use of some of the same measures and techniques as those used for predicting stages of MASLD and MASLD-related cirrhosis for patients undergoing liver biopsy [18]. Hence, machine learning can function along a continuum of disease progression starting early with first MASLD diagnosis. We were somewhat surprised by the finding that FIB-4 was relatively insensitive in predicting mortality. A possible reason for the poor performance of FIB-4 compared to the random forest is the SHAP feature selection utilized early identification of a lower albumin and lower BMI values to predict mortality (See position of blue values for albumin and BMI compared with high red values for age in Fig 2), but the commonly used FIB-4 [7,17] does not incorporate these features to increase the risk factor score. We used XGBoost as a feature selection algorithm given its integration in the SHAP tooling for displaying feature relevance. The use of SHAP was to provide a way to graphically present the importance of variables and consider how such information could be used in a future dashboard for managing patients. Fig 2 shows SHAP values for XGBoost model where the features were selected for use in the RF model. Clinically, hypoalbuminemia is a marker of impaired hepatic synthetic function and poor nutritional status, both associated with adverse outcomes. Similarly, while obesity is a risk factor for MASLD progression, low BMI in this context may reflect sarcopenia or frailty, which are known predictors of poor prognosis in chronic liver disease. A benefit of the random forest method is improved sensitivity and the use of noninvasive scores along with other EHR data as a means of screening for patients with higher risk of mortality. This could provide an improved method to assess which patient would benefit from access to additional resources to track risk of mortality. Liver biopsy is invasive, costly, with the potential risk of complications, so providers currently use various clinical parameters and/or noninvasive tools to help with disease stratification [19,20]. To date however, these tools don’t focus on the earliest point of MASLD identification to determine risk of mortality in a five year window. The random forest models with SHAP feature selection indicate which variables are relevant to identify progression to mortality. Instead of getting arbitrary snapshots of disease severity at points in time, the method uses frequently tracked variables at the earliest point so that patients can be proactively treated.

Consistent with prior reports, we observed that those with HCC were significantly older than those without HCC, and that there were fewer NHB compared with NHW with CIRR [21]. Diabetes is a major risk factor for MASLD progression and is associated with nearly 4-fold increased risk of HCC in patients with MASH cirrhosis [22]. In a real-world study of 18 million European adults, the presence of diabetes was the strongest independent predictor for HCC [23]. In this study, we similarly noted that those with diabetes were more likely to have CIRR, CIRR-HCC, and HCC compared with controls, thus validating the clinical relevance of this study cohort.

The identification of a substantial proportion of patients with HCC but without cirrhosis was remarkable, but consistent with other data [24]. Notably, in an administrative dataset such as the one used here, it is difficult to ascertain whether the non-cirrhotic HCCs developed on a background of early fibrosis (i.e. F1), or whether there was significant or advanced stage fibrosis (i.e. F2-3) where the majority had arisen from livers with advanced fibrosis (i.e. F3). Recent studies from both the VHA and the general population however, have shown that up to 30–40% of those with MASLD-associated HCC may have arisen from a non-cirrhotic liver [24]. Mechanistic studies have also reported that obesity associated oxidative stress may directly modulate HCC development and sarcopenia has been associated with HCC development [25,26]. Therefore, prospective studies with greater representation of the general population will be needed to determine the true prevalence of non-cirrhotic HCCs in the US.

One of the more remarkable findings of this study was that non-Hispanic black Veterans were half as likely to develop cirrhosis associated with MASLD (0.502 CI:0.391–0.644) and one-third as likely to develop cirrhosis and HCC together (0.294 CI:0.128–0.674) as were other populations (Table 2). Additionally, women were one-tenth as likely to develop both cirrhosis and HCC together (0.108 CI:0.015–0.784). These findings may reflect protective biological mechanisms, such as genetic polymorphisms in NHB individuals or the influence of estrogen in women, both of which have been implicated in slower fibrosis progression. Sociodemographic factors, including differences in comorbidity burden, healthcare access, and patterns of screening, may also contribute. However, the exact reasons for these disparities remain unclear and warrant further study in larger, more diverse populations.

Our findings suggest potential clinical applications for predictive models in early management of MASLD. By enabling risk stratification at the time of diagnosis, the model could help identify patients at higher risk of mortality who may benefit from closer follow-up. Our roadmap for future work includes assessment of decision-support dashboards that could support clinicians in real time. In our new VA Merit, we will examine the different outcomes of mortality based on cardiovascular and liver related events along with predicting fibrosis stage. The VA Merit has an assessment of an augmented dashboard to provide longitudinal risk of MASLD progression including survival analysis using Cox proportional hazards models. Hyperparameter tuning of the models will be done in future work to identify how performance can be improved by systematically assessing parameter settings of the models. The dashboard will be assessed by primary care physicians and hepatologists to examine if the information in the predictive models can improve referral of patients to specialty MASLD clinics. Based on a successful phase 3 clinical trial [27], the FDA has approved semaglutide, a glucagon-like peptide-1 (GLP-1), for the treatment of patients with MASH and stage 2 or 3 fibrosis. The MASLD dashboard will be timely to help identify patients who could benefit from the treatment. The earlier patients can be identified, the more options they will have available to them to slow progression of the disease and reduce the risk of mortality associated with MASLD.

For our pilot study, we decided to use as much data as we could for the assessment of RF and FIB-4, which is commonly used to assess severity of MASLD, which resulted in a larger percentage of survivor cases in the test set than in the full cohort. The imbalance could affect the positive predictive values for both RF and FIB-4. In future we will analyze the data with the test set having the same prevalence of death as the full cohort. The cohort was derived from U.S. Veterans, who are older, predominantly male, and more comorbid than the general population, which may limit generalizability. Due to dataset access restrictions and the specificity of our population, external validation could not be performed at this stage. The model is applicable to the VA population having been trained on it, additional work will need to be done to generalize the model outside the VA and have an external validation data set. The team is working on validating the approach on patients in the SSM Health system that treats over eight million patients across four states. While the VA has a 12.6% female population in large cohort studies [28], SSM Health has a 51% female population which makes it ideal to test the generalizability of the approach. Like the VA, SSM Health has a virtual data warehouse for developing and validating predictive models. The system has over five million patients with demographics, labs, vitals, and comorbidities. The effort to demonstrate generalizability through external validation with a more representative population is ongoing work.

Our study is limited by the retrospective design and the use of administrative tools to identify those with MASLD and MASLD-associated complications. For example, the use of elevated liver enzymes likely under-estimated the true population with MASLD. It is also possible that we could have underestimated the number of patients who developed cirrhosis. However, we used previously published data to identify cirrhosis in administrative data [12]. The number of those with HCC could also have been higher had we included codes for HCC interventions such as transarterial chemoembolization or radiofrequency ablation. Another limitation of our study is that we have applied our approach to a specific population that was selected for MASLD. Nevertheless, this cohort was sufficiently large to demonstrate consistency with published reports, [21,23,24] thus validating its utility and findings.

In summary, our data and models demonstrate that information exists in the medical record when an initial diagnosis of MASLD can be made that is associated with the development of cirrhosis and predicts mortality within five years. The random forest model is better than FIB-4 at predicting risk of mortality both early and late in the five years after first diagnosis of MASLD. Prospective studies will be needed to validate such noninvasive machine learning models to predict progression to mortality with MASLD.

## Abbreviations

AAR: Aspartate Aminotransferase and Alanine Aminotransferase Ratio
ALT: Alanine Aminotransferase
ANOVA: Analysis of Variance
AP: Age Platelet Index
APRI: Aspartate Aminotransferase to Platelet Ratio Index
AST: Aspartate Aminotransferase
AUC: Area Under the Curve
BMI: Body Mass Index
EHR: Electronic Health Records
ELF: Enhanced Liver Fibrosis
FIB-4: Fibrosis-4
HBV: Hepatitis B Virus
HCC: Hepatocellular Carcinoma
HCV: Hepatitis C Virus
HEROIC: Health Equity and Rural Outreach Innovation Center
MUSC: Medical University of South Carolina
MASLD: Metabolic Dysfunction Associated Steatotic Liver Disease
MASH: Metabolic Dysfunction Associated Steatohepatitis
NDS: National Data Systems
NFS: : NAFLD Fibrosis Score
NHB: Non-Hispanic Black
NHW: Non-Hispanic White
PCP: Primary Care Providers
ROC: Receiver Operator Characteristic
SE: Standard Error
T2DM: Type 2 Diabetes Mellitus
VA: Veterans Affairs
VIF: Variance Inflation
VINCI: VA Informatics and Computing infrastructure
VIReC: VA Information Resource Center
